# Nusinersen ameliorates motor function and prevents motoneuron Cajal body disassembly and abnormal poly(A) RNA distribution in a SMA mouse model

**DOI:** 10.1038/s41598-020-67569-3

**Published:** 2020-07-01

**Authors:** María T. Berciano, Alba Puente-Bedia, Almudena Medina-Samamé, José C. Rodríguez-Rey, Jordi Calderó, Miguel Lafarga, Olga Tapia

**Affiliations:** 1 Department of Molecular Biology, University of Cantabria, Santander, Spain; 2 Department of Physiology and Pharmacology, University of Cantabria, Santander, Spain; 3 Department of Anatomy and Cell Biology, University of Cantabria, Santander, Spain; 4 “Instituto de Investigación Marqués de Valdecilla” (IDIVAL), Santander, Spain; 5 “Centro de Investigación Biomédica en Red Sobre Enfermedades Neurodegenerativas” (CIBERNED), Madrid, Spain; 6 Department of Experimental Medicine and “Institut de Recerca Biomèdica de Lleida” (IRBLleida), University of Lleida, Lleida, Spain; 7 “Universidad Europea del Atlántico”, Santander, Spain

**Keywords:** Mechanisms of disease, Neurological disorders

## Abstract

Spinal muscular atrophy (SMA) is a devastating autosomal recessive neuromuscular disease characterized by degeneration of spinal cord alpha motor neurons (αMNs). SMA is caused by the homozygous deletion or mutation of the *survival motor neuron 1* (*SMN1*) gene, resulting in reduced expression of SMN protein, which leads to αMN degeneration and muscle atrophy. The majority of transcripts of a second gene (*SMN2*) generate an alternative spliced isoform that lacks exon 7 and produces a truncated nonfunctional form of SMN. A major function of SMN is the biogenesis of spliceosomal snRNPs, which are essential components of the pre-mRNA splicing machinery, the spliceosome. In recent years, new potential therapies have been developed to increase SMN levels, including treatment with antisense oligonucleotides (ASOs). The ASO-nusinersen (Spinraza) promotes the inclusion of exon 7 in *SMN2* transcripts and notably enhances the production of full-length SMN in mouse models of SMA. In this work, we used the intracerebroventricular injection of nusinersen in the SMN∆7 mouse model of SMA to evaluate the effects of this ASO on the behavior of Cajal bodies (CBs), nuclear structures involved in spliceosomal snRNP biogenesis, and the cellular distribution of polyadenylated mRNAs in αMNs. The administration of nusinersen at postnatal day (P) 1 normalized SMN expression in the spinal cord but not in skeletal muscle, rescued the growth curve and improved motor behavior at P12 (late symptomatic stage). Importantly, this ASO recovered the number of canonical CBs in MNs, significantly reduced the abnormal accumulation of polyadenylated RNAs in nuclear granules, and normalized the expression of the pre-mRNAs encoding chondrolectin and choline acetyltransferase, two key factors for αMN homeostasis. We propose that the splicing modulatory function of nusinersen in SMA αMN is mediated by the rescue of CB biogenesis, resulting in enhanced polyadenylated pre-mRNA transcription and splicing and nuclear export of mature mRNAs for translation. Our results support that the selective restoration of SMN expression in the spinal cord has a beneficial impact not only on αMNs but also on skeletal myofibers. However, the rescue of SMN expression in muscle appears to be necessary for the complete recovery of motor function.

## Introduction

Spinal muscular atrophy (SMA) is an autosomal recessive neuromuscular disease that is considered the main genetic cause of infant mortality. SMA is caused by the homozygous loss or mutation of the *survival of motor neuron 1* (*SMN1*) gene, leading to reduced levels of full-length SMN protein. SMN depletion triggers the selective degeneration of lower alpha motor neurons (αMNs), resulting in preferential atrophy of the proximal skeletal muscles with weakness and muscle paralysis^1–3^.

In addition to the *SMN1* gene, humans ubiquitously express one or several copies of a closely related paralog gene called *SMN2*. This gene encodes a full-length SMN protein but presents one consistent difference, that is, the transition C to T (C6T) in exon 7. This change deactivates a splicing enhancer and introduces a splicing silencer, resulting in the exclusion of exon 7 in most *SMN2* transcripts^4,5^. These transcripts are translated into a truncated SMN (SMNΔ7) protein that is rapidly ubiquitylated and degraded by the proteasome system^5–7^. Although the expression of the *SMN2* gene is residual under physiological conditions, the deletion or mutation of *SMN1* confers the number of copies of *SMN2* considerable importance in modifying the severity of SMA phenotypes. Thus, the most severe and frequent SMA is that of type I, which usually has two copies of the *SMN2* gene. Patients with type II or III SMA have a higher copy number of *SMN2*^8–11^.

The functional full-length SMN protein homo-oligomerizes and forms a stable complex (SMN-complex) with Gemin2-8 and Unrip proteins^3,12^. The SMN complex acts as a molecular chaperone to assist the cytoplasmic stage of the assembly of spliceosomal U snRNPs, which are essential splicing factors of the spliceosome^13,14^. Linked to this function of the SMN complex, the Cajal body (CB) is particularly relevant, given its implication in the biogenesis of both spliceosomal U snRNPs and nucleolar snoRNPs^15–18^. In particular, the enrichment of the CB in spliceosomal U snRNPs is consistent with the growing evidence of a spatial association of this organelle with certain transcriptionally active genomic loci of snRNAs^17,19–21^. Moreover, the physical association of CBs with the dense fibrillar component of the nucleolus, the site of synthesis and early processing of pre-rRNAs provides a molecular link for a nucleolus-CB interaction^18,22–24^.

In this context, recent studies from our laboratory have shown that reduced SMN levels in αMNs of a type I SMA patient and the SMN∆7 mouse model induce nuclear features of αMN degeneration, including depletion of canonical CBs, nucleolar stress and altered mRNA processing^25,26^. The dysfunction of mRNA processing in this SMA mouse is also characterized by (i) the early and progressive accumulation of polyadenylated mRNAs in nuclear granules called PARGs (“poly(A) RNA granules”), (ii) the increase in intron-containing pre-mRNAs, and (iii) the disruption of the protein synthesis machinery^25–27^. In fact, the dysfunction of RNA metabolism has enabled us to redefine the SMA as a RNA pathology, although it could also be considered a spliceopathy^28^.

To explore whether normalizing SMN expression in SMN∆7 αMNs during the neonatal period, prior to αMN degeneration, prevents CB depletion and splicing alterations with nuclear retention of unspliced pre-mRNAs in PARGs, we treated SMN∆7 mice with the antisense oligonucleotide (ASO) nusinersen (Spinraza). This 2′-*O*-methoxyethyl (MOE)-modified ASO was designed to increase full-length SMN protein levels by promoting the inclusion of exon 7 in *SMN2* mRNAs. Nusinersen binds to a target site within intron 7, ≅ 10 nucleotides downstream of the 5′-splice site, known as ISS-N1 (intronic splicing silencer N1)^29^. This interaction displaces the splicing suppressor proteins hnRNP A1/A2 and enables spliceosomal U1 snRNPs to bind to the 5′-splice site, thereby promoting the inclusion of exon 7 in *SMN2* transcripts^29–35^. Previous studies in mouse SMA models have demonstrated that nusinersen, when administered directly into the cerebrospinal fluid (CSF), prolongs animal survival and prevents αMN and skeletal muscle pathology^31,36,37^.

Our results in SMN∆7 αMNs show that the intracerebroventricular (ICV) injection of nusinersen at the neonatal period (postnatal day [P] 1) (i) improves motor function, (ii) rescues the CB number, (iii) increases the expression of pre-mRNAs encoding chondrolectin (*Chodl*) and choline acetyltransferase (*Chat*), two key factors for αMN homeostasis^38^, and (iv) reduces the nuclear retention of polyadenylated mRNAs into PARGs at the symptomatic P12 stage. These effects are accompanied by the restoration of the protein synthesis machinery, supporting an effective recovery of mRNA processing and translation.

## Results

### Treatment with ASO-nusinersen (Spinraza) robustly rescues the growth curve, enhances motor function and dramatically reduces αMN loss

Based on previous studies from our laboratory, we have established two temporary windows that demarcate the clinical evolution of SMNΔ7 mice: (i) the preclinical phase (from P0 to P6) and (ii) the clinical phase (from P7 to P12), when the clinical symptoms appear and progress, commonly leading to the death of mice between P12 and P14.

**Figure 1:**
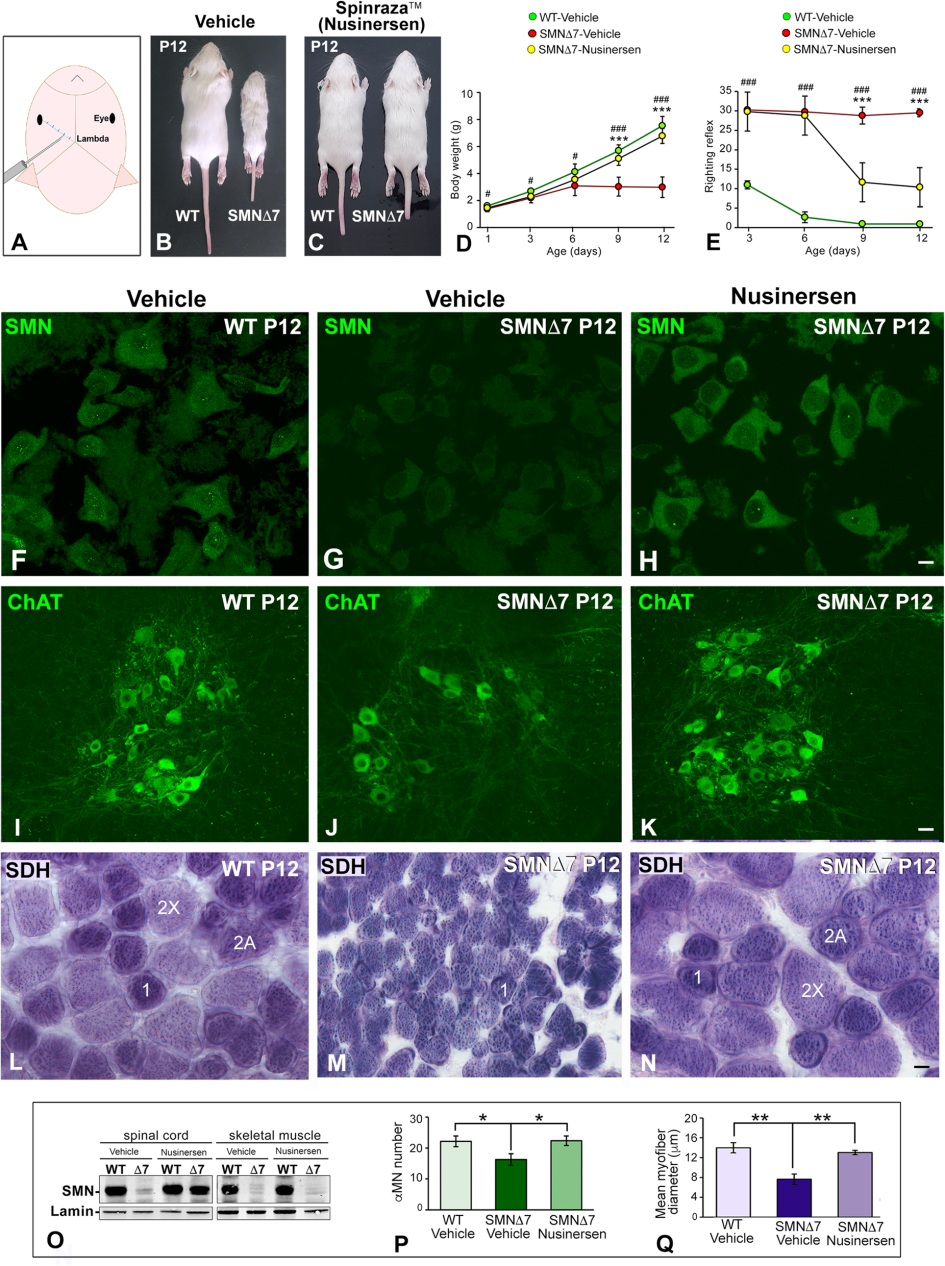
(**A**) Scheme illustrating the intrathecal injection point of the ASO-nusinersen (Spinraza). (**B**,**C**) Images showing differences in body size between WT and SMNΔ7 mice (**B**) and WT and nusinersen-treated SMNΔ7 mice (**C**) at P12. (**D**) Comparative analysis of body weight (mean ± SD) among WT, SMNΔ7 and nusinersen-treated SMNΔ7 mice from P0 to P12 (n = 8 mice per group). ^#^*p* < 0.01, ^###^*p* < 0.0001: WT vs. SMN∆7; ***p < 0.0001: vehicle-treated SMN∆7 vs. nusinersen-treated SMN∆7. (**E**) Response to righting motor reflex acquisition (mean ± SD) at the indicated postnatal periods. Note how nusinersen treatment ameliorates the motor response in SMNΔ7 mice when compared to untreated SMNΔ7 mice (n = 8 mice per group). ^###^*p* < 0.0001: WT vs. SMN∆7; ***p < 0.0001: vehicle-treated SMN∆7 vs. nusinersen-treated SMN∆7. In **C**,**D**, data were analyzed by a one-way ANOVA, followed by the Bonferroni post hoc test using SPSS. (**F**–**H**) Immunodetection of SMN reveals that nusinersen treatment rescues the typical SMN staining pattern in WT αMNs, which appears diffuse in the cytoplasm and concentrated in CBs (**F**,**H**). In contrast, αMNs from the untreated SMNΔ7 mouse exhibit a very weak cytoplasmic SMN signal and CB depletion (**G**). (**I**–**K**) Immunodetection of ChAT in the anterior horn of spinal cord showing how nusinersen treatment increases αMN number in the SMNΔ7 mouse compared with the untreated SMNΔ7 animals. (**L**–**N**) SDH histochemical staining of transverse cryosections of the TA muscle from WT, SMNΔ7 and nusinersen-treated SMNΔ7 mice at P12. In SMNΔ7 mice, this treatment rescues the myofiber phenotype found in WT muscle with a similar distribution of type 1, 2A and 2X myofibers (**L**,**N**). Note that the nusinersen-untreated SMNΔ7 muscle is preferentially composed of smaller type 1 myofibers (**M**). (**O**) Western blot analysis of SMN protein levels in whole spinal cord and skeletal muscle lysates at P12. Note the reduction in SMN levels in the spinal cord and muscle of the SMNΔ7 mouse and the recovery of SMN expression in the spinal cord but not in the muscle upon nusinersen treatment. SMN bands were normalized to lamin, and the relative expression levels of SMN were calculated using ImageJ. Full-length blots are presented in Supplementary Information. (**P**) Quantitative analysis of the number of αMNs (mean ± SD) in transverse cryosections of the spinal cord immunostained for ChAT. αMN counts were performed on the hemispinal cords of WT, SMNΔ7 and nusinersen-treated SMNΔ7 mice at P12. *p < 0.05 when WT and SMN∆7 or vehicle-treated SMN∆7 vs. nusinersen-treated SMN∆7 mice were compared (n = 4 per group, Student’s *t* test analysis was performed using GraphPad). (**Q**) Quantitative analysis of the myofiber diameter (mean ± SD) on transverse cryosections of the TA muscle histochemically stained for SDH detection. Measurements were performed in WT, SMNΔ7 and nusinersen-treated SMNΔ7 mouse muscles at P12. **p < 0.005 when WT and SMN∆7 or vehicle-treated SMN∆7 vs. nusinersen-treated SMN∆7 mice were compared (n = 4 per group, Student’s *t* test analysis was performed using GraphPad). Scale bars: 10 µm (**F**–**H**), 30 µm (**I**–**K**) and 5 µm (**L**–**N**).

The ICV administration of nusinersen (Fig. 1A) at P1 was well tolerated with no obvious signs of toxicity or drug-related death. First, we studied the clinical evolution of SMN∆7 mice treated or not treated with nusinersen. We evaluated the growth curve of mice by performing daily measurement of the body weight from P1 to P12 (Fig. 1C). We noticed that nusinersen treatment had a highly significant impact on body weight gain with a growth curve comparable to that of vehicle-treated WT (hereafter referred to as WT) mice from P6 to P12 (Fig. 1B–D). In fact, nusinersen-treated SMN∆7 mice weighed approximately twice as much as the vehicle-treated mice (hereafter referred to as SMN∆7) at P12 (Fig. 1B–D). On the other hand, nonsignificant differences in body weight were found between WT animals treated with vehicle and nusinersen, indicating that the administration of this ASO does not modify body weight under physiological conditions (“body weight follow-up × genotype”: F_(4,40)_ = 84.23, *p* < 0.0001; “body weight follow-up × nusinersen treatment”: F_(4,40)_ = 57.64, *p* < 0.0001).

Next, we investigated whether nusinersen treatment improves motor functions in SMN∆7 mice by using the righting reflex test. This test evaluates the overall muscle strength and motor coordination, which are severely affected in SMA mice due to weakness of the limb and trunk muscles. The test assesses the time taken for the mouse placed on its back to right itself through 180° to a maximum of 30 s^39^. Whereas the SMNΔ7 mice did not acquire the righting reflex during the neonatal period studied (P1-P12) (Fig. 1E), both WT mice and nusinersen-treated SMNΔ7 mice acquired this motor reflex as early as P3, although the latter showed a temporary delay in the completion of the test (Fig. 1E) (“righting reflex follow-up × genotype”: F_(3,39)_ = 14.85, *p* < 0.0001; “righting reflex follow-up × nusinersen treatment”: F_(3,39)_ = 72.77, *p* < 0.0001). Moreover, whereas SMNΔ7 mice exhibited very severe muscular atrophy, which affects the ability of moving, nusinersen-treated SMNΔ7 mice exhibited moderate hindlimb atrophy and preserved ambulation capacity; however, moderate hindlimb paresis was noticeable in these animals compared to WT mice (see Supplementary Video S1).

To determine whether the clinical improvement of nusinersen-treated SMNΔ7 mice was produced by normalizing SMN levels in the spinal cord, we performed a western blot analysis in tissue lysates at P12. The results in SMNΔ7 mice showed that nusinersen treatment increased SMN expression by 2.5 times in the spinal cord compared with untreated animals. The increase reached approximately 70% of SMN levels from WT samples (Fig. 1O). Next, we investigated whether nusinersen treatment modified SMN protein levels in muscle, a peripheral tissue essentially involved in SMA pathogenesis^40,41^. Western blot analysis of the tibialis anterior (TA) muscle lysates revealed that the neonatal ICV delivery of nusinersen in SMNΔ7 mice did not increase SMN expression in muscle at P12 (Fig. 1O). This result suggests that the therapeutic agent did not cross the blood–brain barrier in neonatal mice, and consequently, the beneficial clinical effect of nusinersen treatment should be mainly due to increased SMN expression in the spinal cord. Consistent with this view, SMN immunolabeling of dissociated αMNs showed that the staining pattern in nusinersen-treated SMNΔ7 neurons was similar to that found in WT neurons. SMN was diffusely distributed in the cytoplasm with a moderate intensity of fluorescent signal. In the nucleus SMN was specifically concentrated in CBs (Fig. 1F,H). Interestingly, αMNs from SMNΔ7 mice exhibited a notably weak cytoplasmic signal of SMN, and CBs were rarely observed (Fig. 1G).

To further understand the effects of nusinersen treatment on the survival of SMNΔ7 αMNs, we performed a quantitative analysis on spinal cord cryosections (lumbosacral region) immunostained for ChAT, a marker of αMNs^40,42^. As expected, nusinersen treatment preserved the population of αMNs in the anterior horn (Fig. 1I,K). Thus, no significant differences in the mean number of αMNs per section were found between WT mice and nusinersen-treated SMNΔ7 animals (Fig. 1P). In contrast, differences were significant when these two groups were compared with SMNΔ7 mice, which had an overt depletion of αMNs (Fig. 1J–K,P).

Since nusinersen treatment prevents αMN death and presumably the formation and maintenance of the neuromuscular junction, we next investigated whether this ASO can prevent muscle atrophy induced by neurogenic myopathy in SMNΔ7 mice. We estimated the myofiber size distribution on cross-cryosections of the TA muscle using the histochemical enzymatic assay for succinate dehydrogenase (SDH), a marker of mitochondrial oxidative activity^40^. Interestingly, despite the reduced SMN expression in the muscle of nusinersen-treated SMNΔ7 mice, nonsignificant differences in myofiber diameter were found between WT mice and nusinersen-treated SMNΔ7 mice at P12 (Fig. 1Q). However, a significant reduction in the myofiber diameter was detected in SMNΔ7 mice compared with nusinersen-treated mice (Fig. 1Q). Moreover, SDH histochemistry revealed that both WT and nusinersen-treated SMNΔ7 myofibers exhibited a staining checkboard pattern of a mixed muscle with a similar proportion of myofibers whose SDH oxidative activity corresponded to type 1, 2A and 2X (Fig. 1L,N). In contrast, vehicle-treated SMNΔ7 myofibers were clearly atrophic with reduced diameter and an oxidative intensity of stain predominantly corresponding to aerobic type 1 myofibers (Fig. 1M,Q). It should be noted that the administration of nusinersen in WT mice did not change the SDH pattern detected in vehicle-treated WT mice (data not shown).

### Nusinersen treatment prevents the disruption and loss of canonical Cajal bodies (CBs) in αMNs of the SMA mouse

The CB is a transcription-dependent nuclear organelle whose number and size accommodate the cellular demands of pre-mRNA splicing^16,18^. Consistent with this notion, our previous studies in αMNs from an SMA patient and the SMNΔ7 mouse have shown that the αMN degeneration caused by SMN deficiency includes an early depletion of canonical CBs and relocalization of coilin as perinucleolar caps and/or within the nucleolus^18,25^. Based on this observation, we considered that the disruption of CBs is an early nuclear sign of αMN degeneration in SMA.

**Figure 2:**
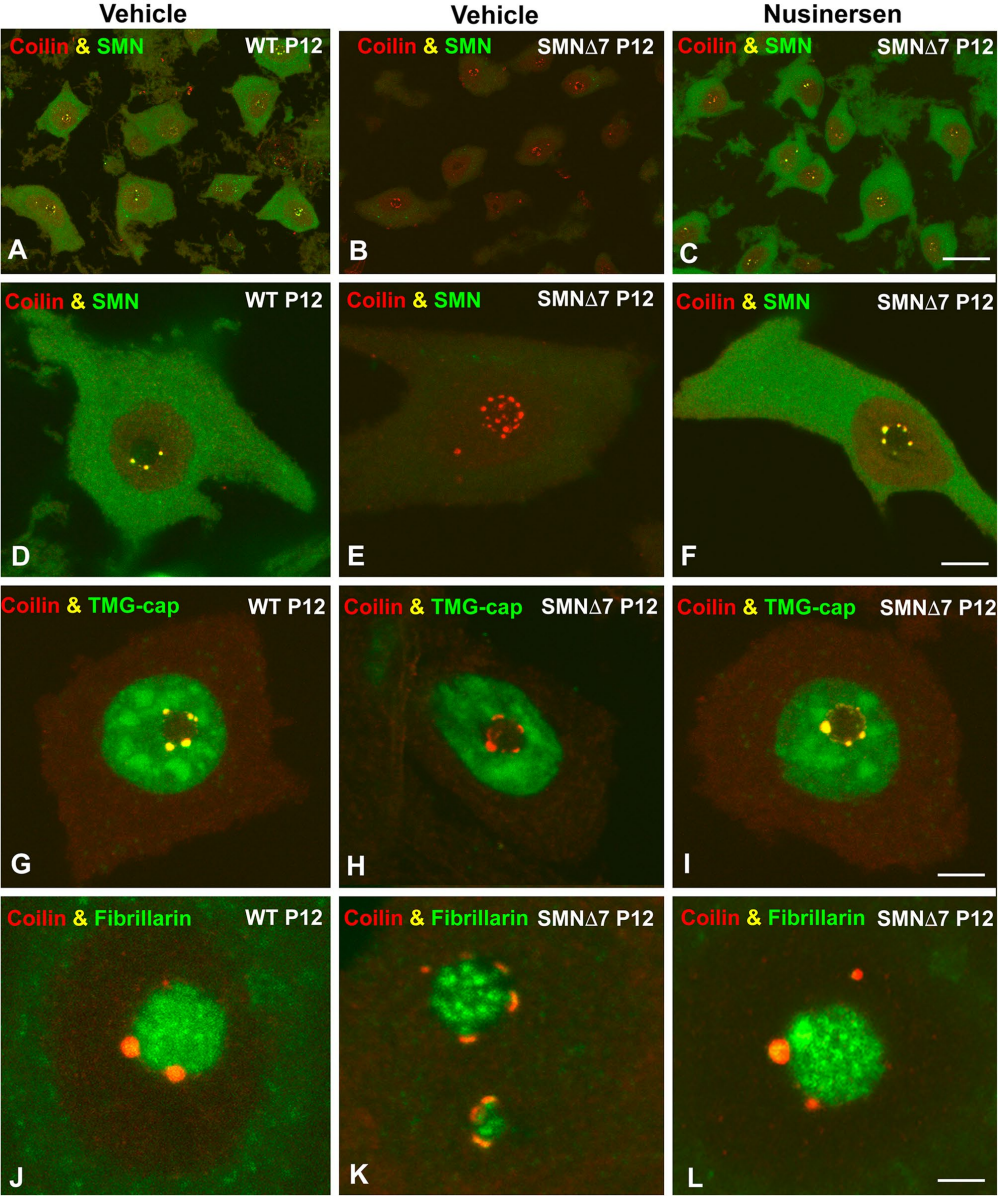
(**A**–**F**) Confocal images of dissociated αMNs from WT (**A**,**D**), SMN∆7 (**B**,**E**) and nusinersen-treated SMN∆7 mice (**C**,**F**) double immunolabeled for coilin (red) and SMN (green). Note that coilin and SMN colocalize in canonical CBs from WT and nusinersen-SMNΔ7 αMNs (**D**,**F**), whereas coilin-positive and SMN-negative perinucleolar caps are detected in αMNs from the vehicle-treated SMNΔ7 mouse. (**G**–**L**) Double immunolabeling for coilin and TMG-cap (**G**–**I**) or fibrillarin (**J**–**L**) in αMNs from WT, SMNΔ7 and nusinersen-treated SMNΔ7 mice. Coilin colocalizes with both TMG-cap and fibrillarin in canonical CBs from WT and nusinersen-treated SMNΔ7 αMNs (**G**,**I**,**J**,**L**). In contrast, the perinucleolar caps of SMNΔ7 αMNs are free from the splicing factor marker TMG-cap (**H**) and exhibit a weak fibrillarin signal (**K**). Scale bars: 20 µm (**A**–**C**), 5 µm (**D**–**I**) and 2.5 µm (**J**–**L**).

Having established that nusinersen treatment preserves the normal population of αMNs and increases SMN protein levels in the spinal cord, we next investigated whether this agent rescues the formation and assembly of canonical CBs in SMNΔ7 αMNs. Double immunolabeling for coilin and SMN revealed the typical cytoplasmic distribution of SMN and the regular presence of canonical CBs, which concentrate coilin and SMN^16,18^, in both WT and nusinersen-treated SMNΔ7 αMNs (Fig. 2A,C,D,F). Moreover, these canonical CBs showed a preferential association with the nucleolus (Fig. 2D,F), as previously reported in other mammalian neurons^18,22^. In contrast, the reduced cytoplasmic SMN staining observed in SMNΔ7 αMNs was accompanied by depletion of canonical CBs and formation of coilin-positive perinucleolar caps, where SMN was not or only slightly detected (Fig. 2B,E). Plots of linear profiles of coilin and SMN fluorescence intensity signals confirmed the colocalization of both molecules in canonical CBs and the absence of SMN in the perinucleolar caps (see Supplementary Fig. S2, A–C).

The neuroprotective effect of nusinersen treatment on SMNΔ7 αMNs was validated by a quantitative analysis of the mean number of CBs per nucleus in neuronal dissociates coimmunostained for coilin and SMN. Nonsignificant differences in the mean number of canonical CBs were detected between WT and nusinersen-treated SMNΔ7 αMNs at P12 (n = 4; 2.44 ± 0.34 vs*.* 2.94 ± 0.54; *p* = 0.18) (Fig. 2A,C). However, in the neurodegenerative phase of SMNΔ7 αMNs (P12), CB number was dramatically reduced compared with WT αMNs (n = 4; 0.08 ± 0.28 in SMNΔ7, vs*.* 2.44 ± 0.34 in WT; *p* = 0.00009) (Fig. 2A,B).

To further characterize the molecular organization of CBs, we performed double immunolabeling for coilin in combination with either TMG-cap, a marker of spliceosomal snRNPs that recognizes the 5′ end of snRNAs^43^, or fibrillarin, a nucleolar snoRNP shared with the CB^18,22^. As expected, both TMG-cap and fibrillarin signals colocalized with coilin in the CBs of WT αMNs and nusinersen-treated SMNΔ7 αMNs (Fig. 2G,I,J,L; Supplementary Fig. S2D, F, G and I), indicating that these nuclear bodies are canonical functional CBs that concentrate splicing factors and snoRNPs in addition to coilin and SMN^16,44^. In contrast, perinucleolar caps of SMNΔ7 αMNs lacked a TMG-cap signal and had weak staining for fibrillarin (Fig. 2H,K).

Finally, it is noteworthy that Gems, coilin-negative and SMN-positive nuclear bodies^45,46^ are conspicuously absent in αMNs in all experimental groups studied.

### Nusinersen treatment rescues the transcription rate of *Chodl* and *ChAT* pre-mRNAs

Previous studies have demonstrated widespread defects in transcription and pre-mRNA splicing of essential genes for αMN homeostasis in cellular and animal models of SMA^27,47–51^. Among these genes, *Chodl* (chondrolectin) and *ChAT* (choline acetyltransferase) are highly expressed in αMNs, playing crucial functions in motor axon growth and neurotransmission, respectively^38,52^. Moreover, Chodl and ChAT are major gene products with dysregulated expression in SMA mouse αMNs^27,38^.

**Figure 3:**
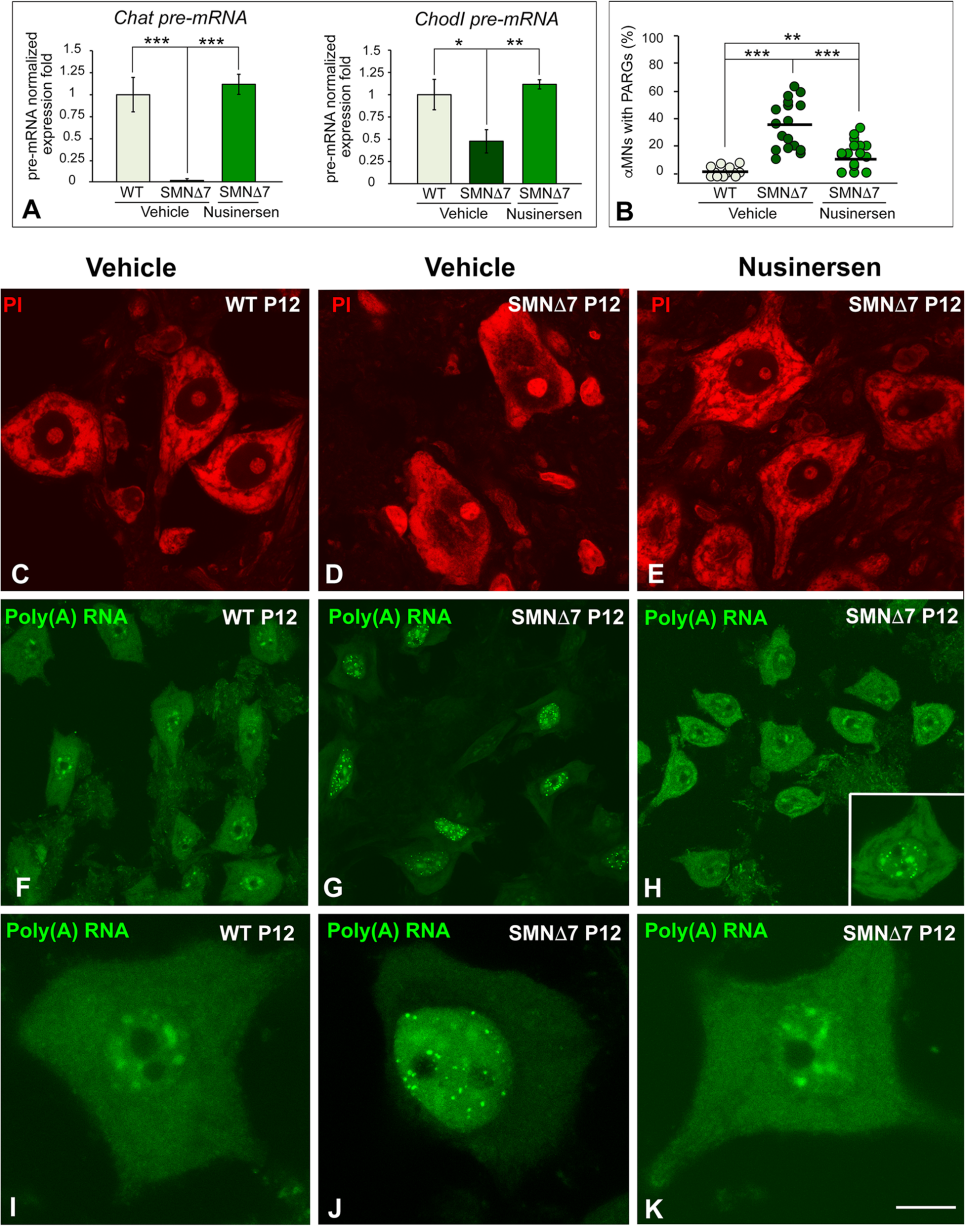
(**A**) RT-PCR analysis of the expression of *Chodl* and *ChAT* pre-mRNAs in whole spinal cord RNA extracts from WT, SMNΔ7 and nusinersen-treated SMNΔ7 mice at P12. Bars represent the mean ± SD, *p < 0.05 and ***p < 0.0005 (n = 4 mice per group, Student’s *t* test analysis was performed using GraphPad). (**B**) The mean percentages of αMNs with PARGs were 1.38% in WT mice and 35.73% in SMN∆7 mice. Nusinersen treatment in SMN∆7 mice significantly reduced the proportion of αMNs with PARGs to 12.04%. *p* values of WT vs*.* SMN∆7 αMNs: 1.78 × 10^–6^ (***p < 0.0005); SMN∆7 vs*.* nusinersen-treated αMNs: 2.8 × 10^–6^ (***p < 0.0005); and WT vs*.* nusinersen-treated SMN∆7: 0.0046 (**p < 0.005) (Student’s *t* test analysis was performed using GraphPad). (**C**–**E**) Dissociated spinal cord αMNs from WT, SMNΔ7 and nusinersen-treated SMNΔ7 mice at P12 stained with propidium iodide (PI) to demonstrate the RNA-rich nucleolus and protein synthesis machinery (Nissl substance). Note the rescue of prominent Nissl bodies in nusinersen-treated SMNΔ7 αMNs (**E**) and the central chromatolysis in untreated SMNΔ7 αMNs (**D**). (**F**–**H**) FISH for poly(A) RNAs in WT, SMNΔ7 and nusinersen-treated SMNΔ7 αMNs at P12. A similar pattern of poly(A) RNA hybridization signal was observed in WT and nusinersen-treated SMNΔ7 αMNs: diffuse in the nucleus, excluding the nucleolus, concentrated in nuclear speckles of splicing factors and diffuse in the cytoplasm (**F**,**H**,**I**,**K**). Note the abundance of nuclear poly(A) RNA-positive granules (PARGs) in numerous SMNΔ7 αMNs, which was accompanied by a substantial reduction of the hybridization signal in the cytoplasm (**G**,**J**). PARGs were occasionally found in nusinersen-treated SMNΔ7 αMNs (**H**, inset). Scale bars: 15 µm (**C**–**E**), 25 µm (**F**–**H**) and 5 µm (**I**–**K**).

To determine whether nusinersen treatment is able to rescue the transcription rate of *Chodl* and *ChAT*, we analyzed the expression levels of both pre-mRNAs by qRT-PCR in spinal cord RNA extracts at P12. We found a significant reduction in *Chodl* and *ChAT* pre-mRNAs in SMNΔ7 samples compared with WT samples (Fig. 3A). Remarkably, nusinersen treatment in SMNΔ7 mice rescued the transcription rates of both genes, which reached pre-mRNA levels comparable to WT ones (Fig. 3A).

### Nusinersen treatment normalizes the distribution of the protein synthesis machinery and polyadenylated mRNAs in SMNΔ7 mice

Our group and others have previously shown a severe perturbation of the protein synthesis machinery with a remarkable nuclear and cytoplasmic redistribution of polyadenylated RNAs and chromatolysis in αMNs of SMA mouse models^26,27,53^. On this basis, we investigated whether nusinersen treatment is able to prevent this severe perturbation of neuronal homeostasis in SMNΔ7 αMNs. Cytochemical staining of RNA-rich structures with propidium iodide revealed prominent Nissl bodies (cytoplasmic areas enriched in rough endoplasmic reticulum and free polyribosomes^54^) distributed throughout the cytoplasm in both WT and nusinersen-treated SMNΔ7 αMNs (Fig. 3C,E). In contrast, most αMNs from SMNΔ7 mice exhibited different degrees of disruption of Nissl bodies, particularly central chromatolysis (Fig. 3D).

Next, we analyzed whether nusinersen treatment preserves the normal distribution of poly(A) RNAs, which include all mRNAs with the exception of histone mRNAs, as an indicator of proper nuclear mRNA processing and export to the cytoplasm. In situ hybridization with the poly-dT probe revealed that poly(A) RNA appeared diffusely distributed throughout the nucleus (excluding the nucleolus), highly concentrated in nuclear speckles and accumulated in extensive cytoplasmic areas of WT αMNs (Fig. 3F,I). In contrast, SMNΔ7 αMNs displayed a prominent nuclear aggregation of poly(A) RNAs in PARGs (Fig. 3G,J), round sharply defined bodies previously reported in mouse SMA αMNs and other neurons under stress conditions^27,55,56^. In addition, we found a cytoplasmic reduction in the poly(A) RNA hybridization signal, suggesting a decreased availability of mRNA for translation (Fig. 3G,J). Interestingly, SMNΔ7 αMNs treated with nusinersen tended to normalize the nuclear and cytoplasmic distribution of polyadenylated mRNAs observed in WT αMNs (Fig. 3H,K). Furthermore, the proportion of αMNs carrying PARGs was significantly reduced in nusinersen-treated SMNΔ7 mice compared to SMNΔ7 animals (Fig. 3B). Since the formation of PARGs begins during the perinatal period^27^, probably before drug administration, we assume that the reduced number of PARGs found in nusinersen-treated SMNΔ7 αMNs is a remnant of the fetal or perinatal stage. We suggest that the nuclear retention of poly(A) RNA into PARGs can be a neuroprotective mechanism to prevent the nuclear export and translation of unprocessed or aberrant polyadenylated mRNAs.

## Discussion

It is well established that αMNs are the main physiopathological cellular targets of SMA, although increasing evidence has revealed the primary importance of other peripheral tissues^41,57,58^. Degeneration of αMNs leads to severe muscular atrophy by denervation, muscle paralysis and premature death by respiratory failure in type I SMA, the most severe form of the disease.

In our previous studies in the SMNΔ7 mouse model of SMA, we observed that SMN depletion in αMNs during the symptomatic postnatal stage severely impacts nuclear compartments involved in pre-rRNA and pre-mRNA processing^26^. Moreover, polyadenylated mRNAs are aggregated in PARGs, reflecting an alteration of mRNA splicing with increased intron retention, abnormal accumulation of incorrectly processed mRNAs and inhibition of the nuclear export of mature mRNAs^27^. This neuronal dysfunction leads to a neurodegenerative process, which is characterized by reduced availability of mature mRNAs for translation, progressive disruption of the protein synthesis machinery and, ultimately, neuronal death^26,27,53^.

In this study, we demonstrate that the therapeutic outcome of the ICV administration of nusinersen (Spinraza) in the SMNΔ7 mouse model of type I SMA is mediated by the selective restoration of SMN levels in the spinal cord but not in muscle. Nusinersen treatment in neonatal SMNΔ7 mice normalizes the growth curve and improves motor function at the late symptomatic stage (P12). These effects are mediated by preserving the normal population of αMNs, as well as the nuclear organization of mRNA processing compartments and the protein synthesis machinery. Consistent with these findings, previous studies in mouse SMA models have demonstrated that nusinersen, when administered directly into the CSF, prolongs animal survival and prevents αMN and skeletal muscle pathology^31,36,37^. However, a recent study using the systemic administration of ASO10-27 in SMA mice supports that peripheral tissues play an essential role in SMA pathology^41^. The authors indicate that the increase in SMN exclusively in peripheral tissues compensates for its deficiency in the spinal cord and preserves αMNs. In our study, the improvements in body weight, motor function and survival in SMN∆7 mice treated with nusinersen were associated with a notable increase in SMN levels in the spinal cord but not in the TA muscle, which exhibited reduced SMN levels, similar to those detected in untreated SMN∆7 mice. Although a fraction of nusinersen injected into cerebral ventricles may be cleared with CSF into the blood and influence peripheral tissues, our results and those from other authors^59^ support that the increase in SMN in the spinal cord is required for the rescue of αMN and skeletal myofiber homeostasis. In fact, we observed that both the skeletal myofiber size distribution and the SDH oxidative staining pattern of myofibers are rescued, despite having reduced SMN levels in muscle. However, the relative importance of SMN restoration in spinal cord versus peripheral tissues in the therapeutic outcome reported in separate studies may be notably influenced by differences in transgenic mouse models of SMA and treatment protocols with ASO.

Consistent with the essential role of nusinersen in the splicing regulation of the *SMN2* gene, promoting the inclusion of exon 7 in the *SMN2* mRNAs and their translation in full-length SMN protein^30–32,34,60^, we detected a recovery in the number and molecular composition of CBs. This neuroprotective mechanism, mediated by the increased expression of SMN in αMNs, can promote the molecular assembly of canonical CBs, which are enriched in coilin, SMN and spliceosomal snRNPs^15,16,43^. CBs are transcription-dependent nuclear organelles involved in spliceosomal snRNP biogenesis required for pre-mRNA splicing^15,16,61^. Depletion of CBs and coilin relocation in the form of perinucleolar caps or within the nucleolus are reliable early signs of neuronal dysfunction in many neurodegenerative disorders, including SMA^18,25,26,62^. It is noteworthy that snRNPs are essential components of the spliceosome, the macromolecular machine for pre-mRNA splicing^14^, and their biogenesis is considerably more efficient in CBs than in the nucleoplasm^63^. In fact, depletion of CBs in cells derived from SMA patients correlates with reduced U4/U6-U5 tri-snRNP assembly, a key maturation step of spliceosomal snRNPs, and with splicing alterations^47,64^. A recent study reported an increase in the number of Gems (SMN-positive and coilin- and snRNP-negative nuclear bodies of unknown function^45,46^) in αMNs following the subcutaneous administration of ASO10-27 in a mouse model of SMA^41^. However, in our study, we have never observed Gems but canonical CBs induced by nusinersen treatment in SMA mice.

In nusinersen-treated SMNΔ7 mice, the transcription rate activation of *Chodl* and *ChAT*, two genes dysregulated in SMA that are essential for αMN survival^38,52^, is consistent with the increase in CBs, whose numbers must be accommodated to the neuronal demand for pre-mRNA processing^44^. In this context, it was also noteworthy that nusinersen treatment in SMNΔ7 mice also rescued the normal organization of the protein synthesis machinery (Nissl bodies) and substantially recovered the nuclear and cytoplasmic compartmentalization of polyadenylated mRNAs. It is well established that all mRNAs, except for histone mRNAs, are polyadenylated in the nucleus, and the poly(A) tail is necessary for efficient mRNA export and translation^65^. The regulation of the nuclear and cytoplasmic compartmented transcriptomes is essential for cell homeostasis. Indeed, in this study and previously^27^, we have demonstrated the nuclear accumulation of polyadenylated RNAs into PARGs, which is associated with intron retention. Nuclear retention of polyadenylated mRNAs may prevent the nuclear export of unspliced or inappropriately expressed transcripts and attenuate translation and energy consumption to maintain energy homeostasis under stress conditions^27,66^. Moreover, nuclear retention of polyadenylated mRNAs may be linked to defective snRNP biogenesis in CBs. In this sense, a recent study^67^ reported that the inhibition of U4 snRNP biogenesis, a key component for U4/U6-U5 tri-snRNP assembly in CBs^68^, results in the abnormal nuclear accumulation of polyadenylated mRNAs. Importantly, the ICV administration of nusinersen in SMN∆7 mice substantially reduced the formation of PARGs and restored the nuclear and cytoplasmic compartmentalization of polyadenylated mRNAs in αMNs. This neuronal response is consistent with a substantial recovery of CB number and pre-mRNA processing and export of mature polyadenylated mRNAs for their translation.

In conclusion, the nusinersen (Spinraza)-dependent rescue of the (i) molecular assembly of canonical CBs, (ii) expression of *Chodl* and *ChAT* primary transcripts, (iii) nuclear and cytoplasmic distribution of polyadenylated mRNAs, and (iv) organization of protein synthesis machinery in αMNs of the SMNΔ7 mouse appears to be essential for maintaining neuronal homeostasis and survival. The increase in SMN expression in the spinal cord partially prevents the severe SMA myopathy induced by αMN degeneration. We suggest that the incomplete recovery of neuromuscular performance in nusinersen-treated SMNΔ7 mice with moderate hindlimb paresis may result from reduced SMN expression in muscle. Further studies will be addressed to investigate the potential role of nusinersen treatment on skeletal myofibers, particularly on the focal disruption of the sarcomere architecture and actin dynamics observed in the SMNΔ7 mice (our unpublished results). In this vein, the combined use of subcutaneous and ICV administration of nusinersen may improve the therapeutic effects of this drug in SMA myopathy^41^.

## Materials and methods

### Animals

*Smn*^+*/−*^*;SMN2*^+*/*+^*;SMN∆7*^+*/*+^*,* heterozygous knockouts for mouse *Smn* (FVB. Cg-Tg[SMN2*delta7]4299Ahmb Tg[SMN2]89Ahmb Smn1tm1Msd/J, stock number 005025), which were purchased from the Jackson Laboratory (Sacramento, USA), were crossed to generate *Smn*^*−/−*^*;SMN2*^+*/*+^*;SMN∆7*^+*/*+^ (SMN∆7) mice and *Smn*^+*/*+^*;SMN2*^+*/*+^*;SMN∆7*^+*/*+^ mice that were wild-type for *Smn* (WT). SMN∆7 mice exhibit a severe postnatal SMA phenotype with a mean lifespan of approximately 2 weeks^69,70^. Animal care and handling were performed in accordance with the Spanish legislation (Spanish Royal Decree 53/2013 BOE) and the guidelines of the European Commission for the Accommodation and Care of Laboratory Animals (revised in Appendix A of the Council Directive 2010/63/UE). The experimental plan was approved by the Ethics Committee of the University of Cantabria and the Committee for Animal Care and Use of the University of Lleida. On P0, the identification of WT and SMN∆7 mice performed by genotyping with PCR. DNA was extracted from the tail, as previously described^69^. Age-matched WT littermates of mutant animals were used as controls.

To investigate the effects of nusinersen (Spinraza) treatment in the αMNs of SMN∆7 mice, we used 32 animals (16 WT mice and 16 SMN∆7 mice) that were divided into four groups. WT and SMN∆7 mice were treated with 10 µl of Spinraza (Biogen) at a dose of 6.5 mg/kg (nusinersen group), or with 10 µl of a 0.9% saline solution (vehicle group). Spinraza or the vehicle was injected at the day of birth (P0) via ICV at the precise point indicated in Fig. 1A using a 10-µl syringe and a custom 33-gauge needle (Hamilton), as described previously^41^. All mice were sacrificed at the late symptomatic neurodegenerative stage (P12)^26,27^.

### Motor righting reflex test

To analyze the effect of nusinersen treatment on motor functions, we used the surface righting test (righting reflex), according to the protocol of Feather-Schussler and Ferguson^39^. Briefly, this test assesses the motor capacity such that a mouse pup can stand up from a supine position. The average age for the rodent straightening reflex to appear is P5. As this test is a reflex, there is no learning component, and it can be repeated throughout the period of experimentation. This test consists of placing the pups face up on a cotton sheet or bench pad and keeping them in this position for 5 s. The pups are then released, and the time it takes to return to the prone position is recorded, as well as the direction of the correction (left or right). A total of 1 min is given for each test, if necessary. The test is repeated for a total of three trials.

### Succinate dehydrogenase (SDH) assay

To investigate the effect of the ICV administration of nusinersen on the phenotype type of TA muscle myofibers in the SMNΔ7 mouse, we used the histochemical SDH assay, a specific mitochondrial marker of myofiber oxidative activity. Thus, most SDH-positive myofibers correspond to small type 1 myofibers, which have slow contraction and high aerobic metabolism. Less SDH-positive myofibers of type 2X of greater caliber are associated with faster contraction and more anaerobic muscle. Finally, type 2A myofibers have an intermediate signal and activity between type 1 and 2X myofibers^71^. Briefly, transverse cryosections were incubated in an SDH reaction mixture (1.5 mM nitroblue tetrazolium, 5 mM EDTA, 48 mM succinic acid, 0.75 mM sodium azide, 30 mM methyl-phenylmethyl sulfate, and phosphate buffered pH 7). During the electron transfer from succinic acid to nitroblue tetrazolium by SDH, a color change occurs, the rate of which represents SDH activity. As controls, we used cryosections incubated in a medium lacking succinic acid. Then, the samples were air-dried and mounted, and images were captured with a microscope (Zeiss, Axioscop Plus) using 40 × or 63 × oil objectives. The morphometric analysis of myofiber diameter was performed on transversal cryosections of the TA muscle stained with the histochemical SDH assay. The measurement of the diameter of each myofiber in the field was performed on randomly collected microscope images using a 40 × objective and ImageJ software (National Institutes of Health). At least 100 myofibers per animal (n = 3, WT; n = 3, SMN∆7; and n = 3, nusinersen-treated SMN∆7 mice) were measured.

### Immunofluorescence and confocal microscopy

For immunofluorescence analysis of αMNs at P12, 4 mice per group (WT, SMN∆7 and nusinersen-treated SMN∆7 mice) were fixed by perfusion with 3.7% paraformaldehyde (freshly prepared) in phosphate-buffered saline (PBS) under deep anesthesia with pentobarbital (50 mg/kg). The spinal cords were rapidly dissected, removed, postfixed for 6 h and washed in PBS. Transverse sections (160-µm thick) of the spinal cord were obtained with a vibratome, and small tissue fragments from the anterior horn were dissected out. The samples were transferred to a drop of PBS on a positively charged slide (Superfrost Plus, Thermo Scientific, Germany), and squash preparations of dissociated MNs were generated following the previously reported procedure^43^.

For muscle, tissue fragments of the TA were pinned to cork, immersed in ice-cold relaxing buffer (100 mM NaCl, 2 mM KCl, 2 mM MgCl_2_, 6 mM K_3_PO_4_, 1 mM EGTA, and 0.1% glucose; pH 7.0) and relaxed at 4 °C until fixed in 3.7% paraformaldehyde^72^. Muscle fragments were cryoprotected in 15% and 30% sucrose in PBS (phosphate saline buffer: 137 mM NaCl, 2.7 mM KCl, 8 mM Na_2_HPO_4_, and 2 mM KH_2_PO_4_; pH 7.4) until they sank, embedded in Tissue-Tek OCT Compound (Sakura FineTek USA), and frozen on a chilled metal block in dry ice. Cryosections measuring 8 μm in thickness were mounted on SuperFrost slides and stored at − 80 °C until use.

For immunofluorescence, all samples were processed as described in Pena et al.^43^. Briefly, samples were treated with 0.5% Triton X-100 for 45 min and incubated for 3 h with the primary antibody containing at room temperature. Specific secondary antibody conjugated with FITC, Texas Red or Cy3, or Cy5 (diluted 1:75, Jackson, USA) were used. Some samples were counterstained with propidium iodide (PI) or Phalloidin-FITC. The slides were mounted with ProLong Anti-Fading Medium (Invitrogen).

We used an LSM510 (Zeiss, Germany) laser scanning microscope using a 63 × oil (1.4 NA) objective. To avoid overlapping signals, images were obtained by sequential excitation at 488 nm and 543 nm. The fluorescence profiles of confocal intensity signals across a line shown in Fig. S2 were generated using confocal images of double immunolabeled samples. Images were processed using Adobe Photoshop CS6 software.

The following primary antibodies were used for immunofluorescence: mouse monoclonal anti-SMN (diluted 1:100, catalog no. 610646, BD Transduction Laboratories, USA); mouse monoclonal anti-TMG-cap (NA02A, Oncogene, USA); mouse monoclonal anti-fibrillarin (diluted 1:100, catalog no. ab4566, Abcam, USA); rabbit polyclonal anti-coilin (1:500, 204.10, Prof. A. Lamond, Dundee, UK); and goat polyclonal anti-choline acetyltransferase (ChAT) (1:200, AB144P, Millipore, MA, USA).

### In situ hybridization and quantification

Fluorescence in situ hybridization (FISH) was performed according to the protocol described by Narcís et al.^27^. Preparations of MNs were permeabilized with TBS-E-SDS for 15 min at 37 °C, washed three times in 6 × SSPE-0.1% Tween 20 for 15 min, and incubated with the probe containing tRNA for 3 h at 42 °C in a humidified chamber. An oligo(dT)_(50)_-mer, 5′-end-labeled with biotin (MWG-Biotech, Germany), was used as a probe for fluorescence in situ hybridization (FISH) to poly(A) RNA. The hybridization mixture contained 80 ng of oligo dT(50), 2 × SSC, 1 mg/ml tRNA, 10% dextran sulfate and 25% formamide. After hybridization, preparations of MNs were washed in 6 × SSC for 15 min and then washed in 4 × SSC-0.1% Tween 20 for 15 min at room temperature. The hybridization signal was detected with FITC-avidin for 30 min. All samples were mounted with Vectashield (Vector, USA).

The quantitative analysis of the proportion of SMN∆7 αMNs containing PARGs was performed in squash preparations processed for FISH with the poly(dT) probe. The proportion of neurons containing these granules was estimated by direct examination of the different focal planes throughout neuronal nuclei using a 40 × objective. Quantification was performed on at least 100 αMNs from WT (n = 4), SMN∆7 (n = 4) and nusinersen-treated SMN∆7 (n = 4) mice.

### qRT-PCR for relative gene expression analysis

For qRT-PCR, we used WT (n = 4), SMN∆7 (n = 4) and nusinersen-treated SMN∆7 (n = 4) mice. Mice were decapitated after being anesthetized, and the lumbar spinal cord was quickly removed and frozen in liquid nitrogen. RNA was isolated with TRIzol following the manufacturer’s instructions (Invitrogen, Carlsbad) and purified with the RNeasy kit (Qiagen, Hilden, Germany).

According to our laboratory protocols^27^, 1 μg of RNA was reverse-transcribed to first-strand cDNA utilizing a High Capacity cDNA Reverse Transcription Kit (Life Technologies) using random hexamers as primers. The expression of the mRNA candidates *ChAT* and *Chodl* was determined by qRT-PCR using gene-specific SYBR Green-based primers (Invitrogen). The threshold cycle (Ct) for each well was determined and the results were normalized to *Gapdh* mRNA. Relative gene expression was calculated according to the 2-(ΔΔCt) equation^73^. Each value in this work represents the mean ± SD of independent samples obtained under the same conditions and compared to two replicated qRT-PCR analyses. The SYBR Green-based specific primers for murine pre-RNAs analyzed were as follows: for the pre-mRNA of *ChAT* 5′-CTTGGGGCCAGTCTGATAGC-3′ and 5′-GGACACATGGCTAGAAGGGG-3′ and for the pre-mRNA of *Chodl* 5′-GCTGTTGTCTCCCGCATCTT-3′ and 5′-AAGTGGAAGCGTTTGGGATT-3′.

### SDS-PAGE and immunoblotting

Analysis of the level of SMN protein were performed according to the protocol described by Narcís et al. ^27^. Briefly, spinal cords and skeletal TA muscle samples from WT (n = 3), SMN∆7 (n = 3) and nusinersen-treated SMN∆7 (n = 4) mice were lysed at 4 °C in a buffer containing 50 mM Tris (pH 8), 150 mM NaCl, 2% Nonidet NP-40, 1 mM MgCl_2_, 1 mM dithiothreitol, and 10% glycerol and supplemented with EDTA-free complete protease inhibitor cocktail and PhosphoSTOP (Roche). Samples were sonicated and cleared by centrifugation at 14,000 rpm for 10 min at 4 °C. The proteins were separated on 4–20% NuPage TG SDS–PAGE gels (Invitrogen) and transferred to nitrocellulose membranes using standard procedures. Mouse monoclonal anti-SMN (diluted 1:500) and rabbit polyclonal anti-Lamin A/C (diluted 1:1,000, generously donated by Prof. Gerace) were used. Protein bands were detected with an Odyssey Infrared-Imaging System (Li-Cor Biosciences) according to the Odyssey Western-Blotting Protocol. For the quantitative analysis of the blots, ImageJ software was used (U.S. National Institutes of Health, Bethesda, Maryland, USA, https://imagej.nih.gov/ij).

### Statistical analysis

For comparisons among the experimental groups, data were analyzed using GraphPad Prism 7 software and PASW Statistics 22 (SPSS, Inc.) packages. Data are expressed as mean ± SD. The statistical analysis was assessed by one-way ANOVA or mixed-design two-way ANOVA (Split-Plot ANOVA), followed by the Bonferroni post hoc test (SPSS) or Student’s *t* test (GraphPad). In all cases, differences were considered to be statistically significant if *p* < 0.05.

## Supplementary information

Supplementary information 1

Supplementary information 2

Supplementary video
