# Supplementary material for: Nusinersen ameliorates motor function and prevents motoneuron Cajal body disassembly and abnormal poly(A) RNA distribution in a SMA mouse model
Source: Sci Rep. 2020 Jul 1;10:10738. doi: 10.1038/s41598-020-67569-3 (PMC7330045; doi:10.1038/s41598-020-67569-3)
Supplement: Supplementary file 1 — Supplementary information 1 [file 41598_2020_67569_MOESM1_ESM.pdf]

Nusinersen ameliorates motor function and prevents motoneuron Cajal body disassembly and abnormal poly(A) RNA distribution in a SMA mouse model.

María T. Berciano[1,4,5*], Alba Puente-Bedia[2*], Almudena Medina-Samamé[3], José C. Rodríguez-Rey[1,4], Jordi Calderó[6], Miguel Lafarga[3,4,5] and Olga Tapia[4,5,7]

1. Department of Molecular Biology, University of Cantabria, Santander, Spain.

2. Department of Physiology and Pharmacology, University of Cantabria, Santander, Spain.

3. Department of Anatomy and Cell Biology, University of Cantabria, Santander, Spain.

4. *Instituto de Investigación Marqués de Valdecilla* IDIVAL, Santander, Spain

5. Centro de Investigación Biomédica en Red sobre Enfermedades Neurodegenerativas (CIBERNED).

6. Department of Experimental Medicine, School of Medicine, University of Lleida and *Institut de Recerca Biomèdica de Lleida* (IRBLleida), Lleida, Spain.

7. *Universidad Europea del Atlántico*, Santander, Spain.

* Contributed equally

Abbreviated title: Effects of nusinersen in SMA motor neurons

Corresponding author:

Dr. Olga Tapia
Instituto de Investigación Marqués de Valdecilla IDIVAL
Av. Cardenal Herrera Oria s/n
39011 Santander, Spain
e-mail: tapiao@unican.es

Supplemental Information

Supplementary Video S1.

See attached file Video S1.mp4

Supplementary Video S1. By P12, important variations were noted among the 3 experimental groups (WT, SMNΔ7 and nusinersen-treated SMNΔ7 mice). The attached video illustrates 3 comparable mice at P12 (each of one representing the 50th percentile of its group). It can be noted that SMNΔ7 mice were significantly smaller in size than nusinersen-treated SMNΔ7 mice, which have almost the same size as WT mice. Regarding motor performance, important differences were also noted. Whereas SMNΔ7 mice exhibited a very severe amyotrophy that conditioned the inability to move, mice treated with nusinersen had moderate hindlimb muscle atrophy and preserved their ambulation capacity. Within this group, moderate hindlimb paresis was also noticeable in comparison with the WT group.

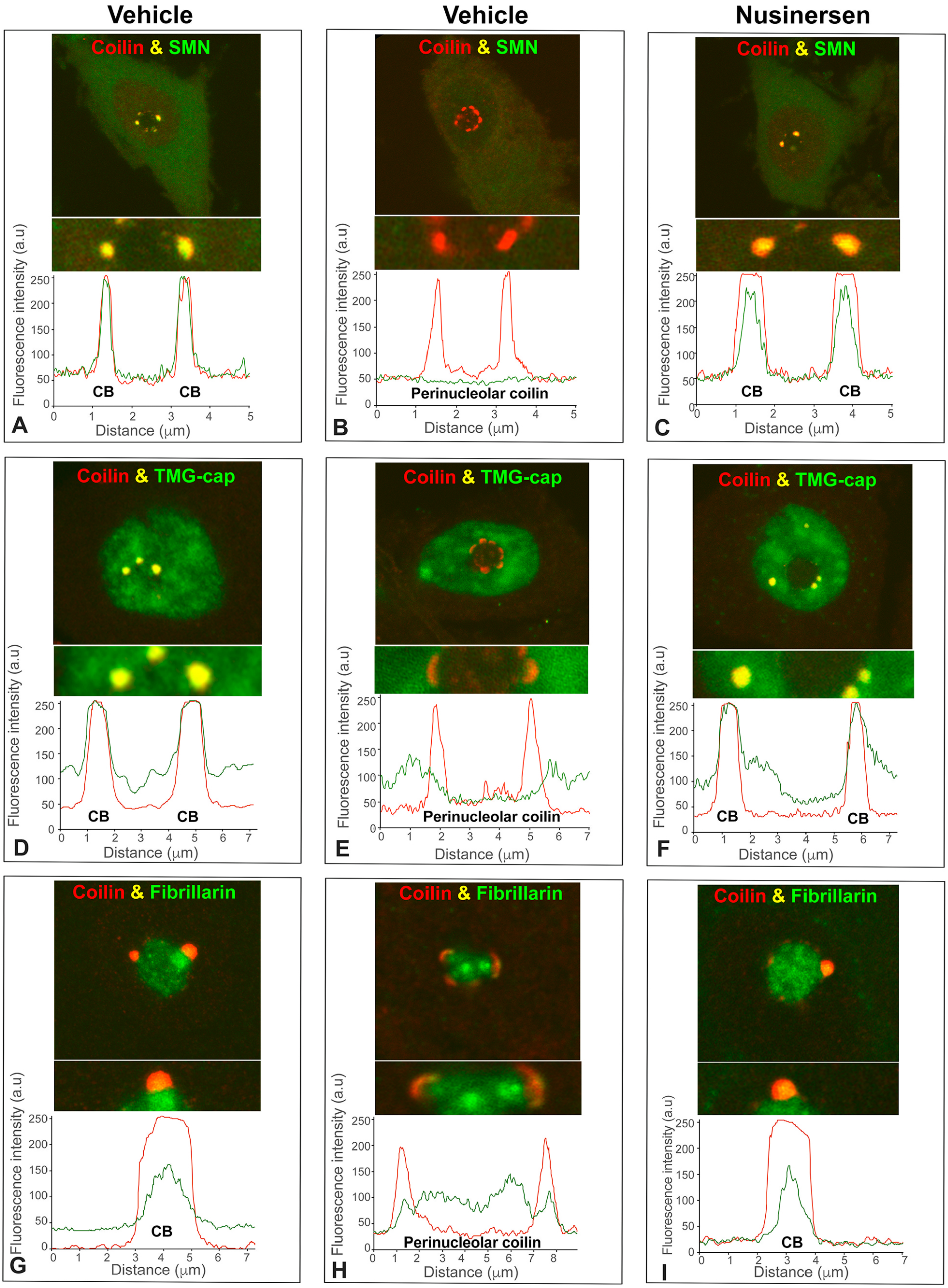

Supplementary Figure S2. Linear profiles of the fluorescence signal intensities of coilin and SMN (A-C), coilin and TMG-cap (D-F), and coilin and fibrillarin (G-I) in WT, SMNΔ7 and nusinersen-treated SMNΔ7 αMNs. Note the colocalization of coilin with SMN, TMG-cap and fibrillarin in canonical CBs from WT and nusinersen-treated SMNΔ7 αMNs, as well as the absence of both SMN and TMG-cap in perinucleolar caps of SMNΔ7 αMNs.
