# Supplementary material for: Nusinersen ameliorates motor function and prevents motoneuron Cajal body disassembly and abnormal poly(A) RNA distribution in a SMA mouse model
Source: Sci Rep. 2020 Jul 1;10:10738. doi: 10.1038/s41598-020-67569-3 (PMC7330045; doi:10.1038/s41598-020-67569-3)

Nusinersen ameliorates motor function and prevents motoneuron Cajal body disassembly and abnormal poly(A) RNA distribution in a SMA mouse model.

María T. Berciano[1,4,5*], Alba Puente-Bedia[2*], Almudena Medina-Samamé[3], José C. Rodríguez-Rey[1,4], Jordi Calderó[6], Miguel Lafarga[3,4,5] and Olga Tapia[4,5,7]

1. Department of Molecular Biology, University of Cantabria, Santander, Spain.
2. Department of Physiology and Pharmacology, University of Cantabria, Santander, Spain.
3. Department of Anatomy and Cell Biology, University of Cantabria, Santander, Spain.
4. *Instituto de Investigación Marqués de Valdecilla* IDIVAL, Santander, Spain
5. *Centro de Investigación Biomédica en Red sobre Enfermedades Neurodegenerativas* (CIBERNED).
6. Department of Experimental Medicine, School of Medicine, University of Lleida and *Institut de Recerca Biomèdica de Lleida* (IRBLleida), Lleida, Spain.
7. *Universidad Europea del Atlántico*, Santander, Spain.

* Contributed equally

Abbreviated title: Effects of nusinersen in SMA motor neurons

Corresponding author:

Dr. Olga Tapia
Instituto de Investigación Marqués de Valdecilla IDIVAL
Av. Cardenal Herrera Oria s/n
39011 Santander, Spain
e-mail: tapiao@unican.es

Supplemental Information. Full-lenght blots corresponding to Fig. 1O are presented

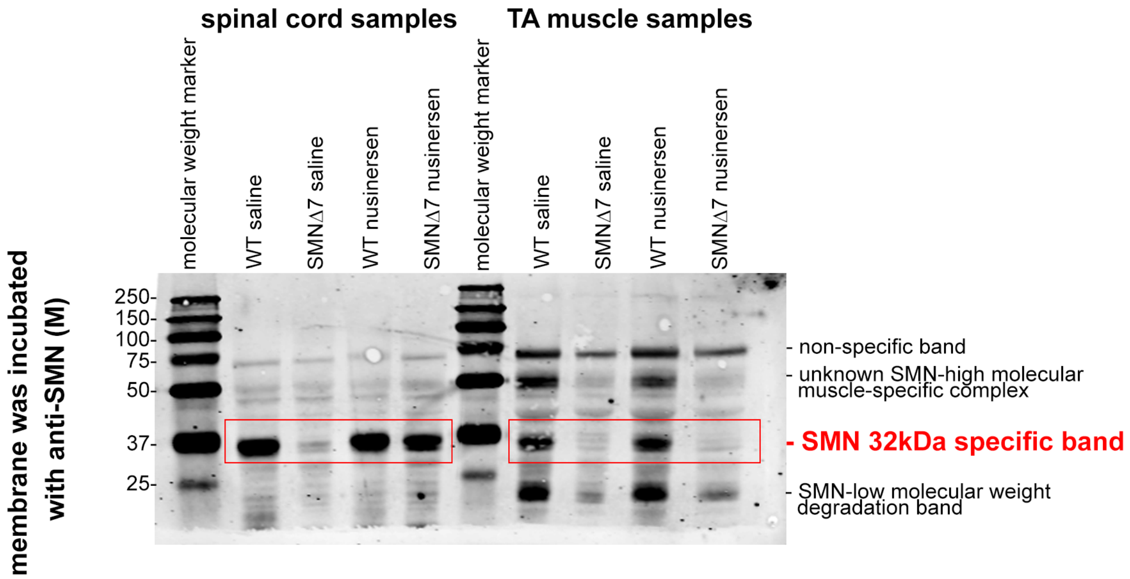

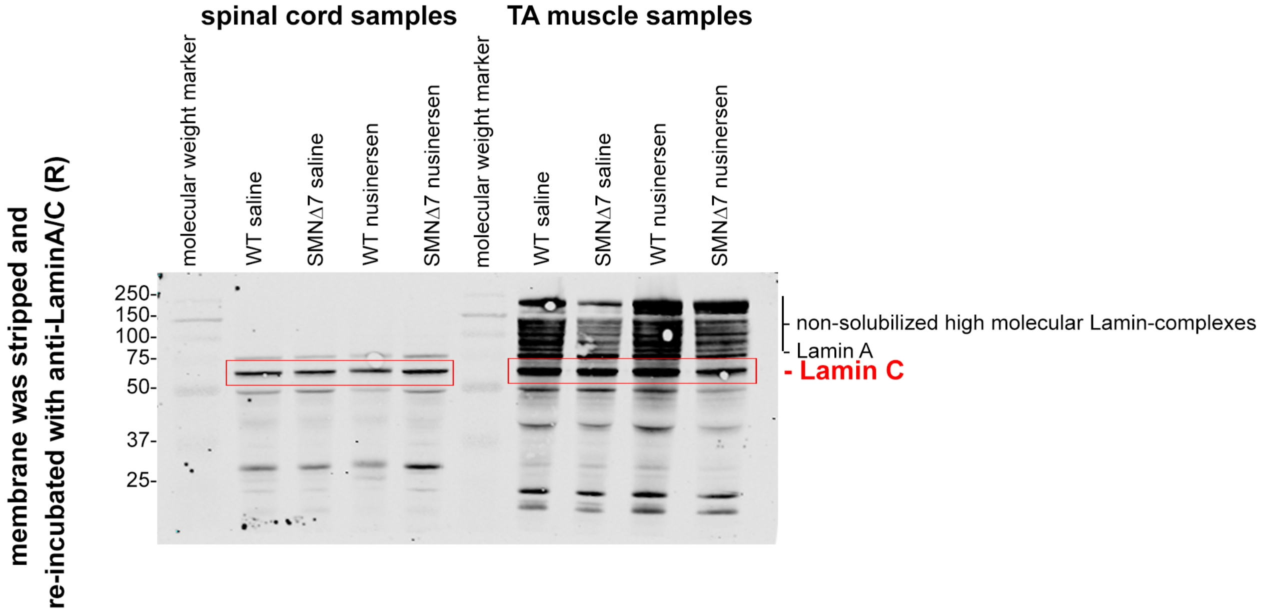

Supplement: Supplementary file 2 — Supplementary information 2 [file 41598_2020_67569_MOESM2_ESM.pdf]
